# From Key Encapsulation to Authenticated Group Key Establishment—A Compiler for Post-Quantum Primitives †

**DOI:** 10.3390/e21121183

**Published:** 2019-11-30

**Authors:** Edoardo Persichetti, Rainer Steinwandt, Adriana Suárez Corona

**Affiliations:** 1 Department of Mathematical Sciences, Florida Atlantic University, Boca Raton, FL 33431, USA; epersichetti@fau.edu (E.P.); rsteinwa@fau.edu (R.S.); 2 Department of Mathematical Sciences, Universidad de León, 24071 León, Spain

**Keywords:** authenticated group key establishment, post-quantum cryptography, key encapsulation mechanism

## Abstract

Assuming the availability of an existentially unforgeable signature scheme and an (IND- CCA secure) key encapsulation mechanism, we present a generic construction for group key establishment. The construction is designed with existing proposals for post-quantum cryptography in mind. Applied with such existing proposals and assuming their security, we obtain a quantum-safe three-round protocol for authenticated group key establishment that requires only one signature per protocol participant.

## 1. Introduction

To enable confidential communication among a group of two or more users over an insecure network, cryptography provides group key establishment procotols. Dealing with a non-trusted communication infrastructure, the question of authenticating protocol participants naturally arises in this context as well. The resulting cryptographic protocols are commonly application-agnostic and focus only on the task of establishing a shared high-entropy secret among the legitimate users, not mandating any particular usage of that secret key in subsequent applications. The resulting task, authenticated group key establishment (AGKE), is fairly well-understood, even though there is a remarkable diversity in the details of security models in use. A standard technique to derive an AGKE solution is to apply some form of protocol compiler or generic framework to a passively secure solution. If a public-key infrastructure is available, signatures provide an adequate mechanism. This results commonly in protocols with a substantial number of signatures being computed, transmitted, and verified:

- In the Katz-Yung compiler [1], for each message sent in the original protocol, a signature has to be computed and transmitted (and verified). For instance, Apon et al. [2] propose the application of this compiler for their unauthenticated group key establishment solution.
- The compiler made by Bresson et al., C-AMA [3] requires a signature for each message in the original protocol, plus one more for each protocol participant (and according signature verifications).
- Bohli’s framework for robust group key agreement [4] targets two-round protocols, and in each round each participant sends a signed message (and verifies signatures by all other participants).

A popular AGKE building block is the traditional Diffie–Hellman two-party key exchange. This one-round protocol for two parties enables elegant two-round solutions for group key establishment—the Burmester–Desmedt protocol [5] offering a prominent design. Using a compiler (or a tailored design approach), deriving an AGKE solution with two or three rounds has by now become standard practice. Regrettably, owing to Shor’s algorithm for solving discrete logarithms [6], the traditional Diffie–Hellman protocol is no longer a viable building block for post-quantum AGKE. While the cryptanalytic threat of quantum computers should not be overstated (cf., e.g., [7,8]), in view of steady technological progress, it is, therefore, necessary to provide new solutions that will be robust in the post-quantum setting.

With the current status of quantum cryptanalysis, it is clear that mathematical tools relying on the hardness of factoring or computing discrete logarithms are no longer an option. Regrettably, no “silver bullet” is known that would allow a seamless replacement of today’s solutions with quantum-safe ones. In this scenario, the cost of integrating signatures in a protocol can differ quite a bit from the familiar setting. Specifically, for hash-based designs, arguably one of the most popular approaches for post-quantum signing, the size of signatures remains an issue. For instance, proposed instances of the SPHINCS${}^{+}$ design have signature lengths between 8080 and 49,216 bytes [9]. Taking this into account, a compiler by Tang and Mitchell [10] appears more attractive from today’s post-quantum perspective. Assuming the a priori availability of a unique session identifier that is distributed among the protocol participants, Tang and Mitchell present a compiler where each participant computes and transmits only one signature (and performs signature verifications).

### Our Contribution

With the current state of the the art, key encapsulation mechanisms (KEMs) are a natural starting point for implementing key establishment solutions in a post-quantum setting. This is evident, for example, when looking at the ongoing NIST standardization effort [11], where the vast majority of candidates for public-key encryption and key exchange are indeed presented as KEMs. In this paper, we show how to derive a three-round AGKE using KEMs, where no participant signs more than one message. This is one round less than applying the Katz–Yung compiler to Apon et al.’s unauthenticated three-round protocol [2], and differing from the Katz–Yung-based construction, our approach requires only one signature per protocol participant. The signature scheme we use is assumed to be EUF-CMA secure, and the KEM we involve is assumed to be IND-CCA secure.

## 2. Preliminaries

Here and in the subsequent sections, the security parameter will be denoted by *ℓ*, and notions like polynomial time or negligible refer to that parameter.

### 2.1. Key Encapsulation Mechanism

A main technical tool in our construction are key encapsulation mechanisms (introduced by Shoup in [12]), which are a structure similar to public-key encryption schemes, with the main difference being that the goal is to encrypt (“encapsulate”) a randomly-generated symmetric key. A formal definition is as follows.

**Definition** **1** (Key Encapsulation Mechanism)**.** *A key encapsulation mechanism (KEM) is a triple of polynomial time algorithms (KeyGen,Encaps,Decaps) as follows:*

KeyGen *is probabilistic. Given the security parameter ℓ, it generates a pair of public and secret keys (pk,sk).*
Encaps *is probabilistic. Given a public key pk, it generates a pair (K,C) where $K\in {\left\{0,1\right\}}^{\ell }$ is a symmetric key and C is an encapsulation of this key under the public key pk.*
Decaps *is deterministic. Given a secret key sk and an encapsulation C, this algorithm outputs the symmetric key K or a special error symbol *⊥*.*

For our purposes, the KEM needs to be perfectly correct, i.e., we require that for all key pairs (pk,sk) generated by KeyGen the following correctness condition holds: if (K,C) is an output of Encaps(pk), then ${\mathrm{Decaps}}_{sk}\left(C\right)=K$.

The most relevant notion to capture the security of a KEM is that of indistinguishability under chosen-ciphertext attacks (also defined in [12]), which is once again similar to the usual one for PKEs, except that the adversary is now asked to distinguish between an honestly encapsulated key and a pseudorandom value. We provide a formal definition below.

**Figure 1:**
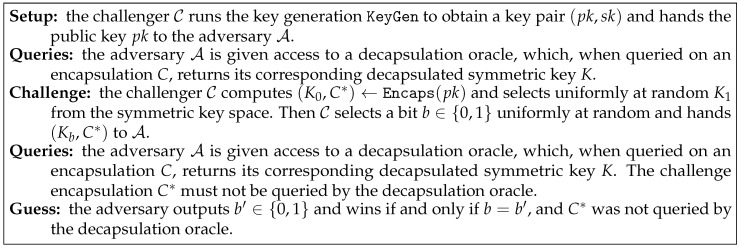
IND-CCA security of a key encapsulation mechanism.

**Definition** **2** (IND-CCA Security)**.** *A KEM is IND-CCA secure if the advantage of any probabilistic polynomial time adversary A in the game described in Figure 1, as a function in the security parameter, is negligible. Here the advantage of an adversary A is defined as ${\mathrm{Adv}}_{A}^{\mathrm{IND}-\mathrm{CCA}}\left(\ell \right)=\left|2\cdot \mathrm{Pr}\left[b=b^{\prime }\right]-1\right|$.*

### 2.2. Signature Scheme

To formalize a signature scheme, we follow the usual approach.

**Definition** **3** (Signature)**.** *A signature is a triple of polynomial time algorithms (SigKeyGen,Sign,Verify) as follows:*

- SigKeyGen *is probabilistic. Given the security parameter ℓ, it generates a pair of public and secret keys (vk,sigk).*
- Sign *is probabilistic. Given a secret key sigk and a message M it generates a signature σ.*
- Verify *is deterministic. Given a public key vk, a signature σ, and a message M, this algorithm outputs *1* if the signature is valid and *0* otherwise.*

*We require that for all key pairs (vk,sigk) generated by* SigKeyGen *and for all messages M the following correctness condition holds: If σ is an output of Sign(sigk,M), then ${\mathrm{Verify}}_{vk}\left(\sigma ,M\right)=1$.*

The security notion usually required for signatures is existential unforgeability under chosen message attacks, and we capture it by the following definition.

**Figure 2:**
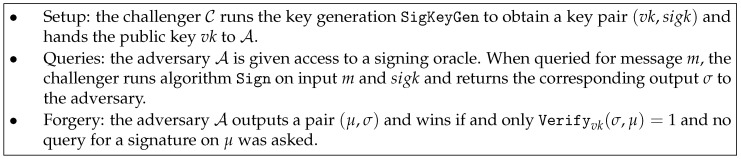
EUF-CMA security of a signature scheme.

**Definition** **4** (EUF-CMA security)**.** *A signature scheme is EUF-CMA secure if the advantage of any probabilistic polynomial time adversary A in the game described in Figure 2 is negligible. Here the advantage of an adversary A is defined as the function ${\mathrm{Adv}}_{A}^{\mathrm{EUF}-\mathrm{CMA}}\left(\ell \right)=\mathrm{Pr}\left[{\mathrm{Succ}}_{A}^{\mathrm{EUF}-\mathrm{CMA}}\right]$, where ${\mathrm{Succ}}_{A}^{\mathrm{EUF}-\mathrm{CMA}}$ is the event that A wins.*

## 3. Security Model and Security Goals

The security model we use follows the oracle-based approach to capture adversarial capabilities—this approach is also used by Katz and Yung [1], for instance, building on the work of Bresson et al., work [13].

### 3.1. Protocol Participants

Given a security parameter *ℓ*, we assume that the set of protocol participants U is polynomial in *ℓ*. We model each user U∈U as a probabilistic polynomial time algorithm which is allowed to execute a polynomial number of protocol instances $\Pi_{U}^{s}$ (s∈N) concurrently. We assume user identities to be bitstrings of identical length *ℓ* and to keep notation simple, throughout we will use *U* referring to both the bitstring identifying a user *U* and the algorithm *U* itself. Let $\Pi_{U}^{s}$ be a protocol instance, the following seven variables are associated with it:

${\mathrm{pid}}_{U}^{s}$: stores the identities of those users in U with which a key is to be established a particular instance aims at establishing a key with, including *U* (this ensures being partnered is thus a reflexive relation);
${\mathrm{sid}}_{U}^{s}$: stores a session identifier, i.e., a non-secret public identifier for the session key ${\mathrm{sk}}_{U}^{s}$;
${\mathrm{sk}}_{U}^{s}$: stores a distinguished null value and after a successful protocol run holds the session key;
${\mathrm{acc}}_{U}^{s}$: is set to true if the session key stored in ${\mathrm{sk}}_{U}^{s}$ has been accepted;
${\mathrm{state}}_{U}^{s}$: keeps state information needed while executing the protocol (e.g., the secret scalars used as ephemeral Diffie–Hellman keys);
${\mathrm{term}}_{U}^{s}$: is set to true if this protocol execution has terminated;
${\mathrm{used}}_{U}^{s}$: indicates if this instance is used, i.e., currently involved in a protocol execution.

### 3.2. Initialization

Before actual protocol executions take place, we allow an optional trusted initialization phase without adversarial interference. During this phase, for each user public and private key pairs $\left(pk_{U},ak_{U}\right)$ can be generated and distributed accordingly. These private and public keys can include the ones corresponding to a KEM, a signature scheme, etc. The initialization phase can also be used to issue (possibly needed) further public parameters.

### 3.3. Adversarial Capabilities and Communication Network

The adversary A is represented as a probabilistic polynomial time algorithm with full control over the communication network. The network is, therefore, fully asynchronous, non-private, allowing arbitrary point-to-point connections among users. More specifically, we express the capabilities of the adversary through the following oracles:

Send(U,s,M): This oracle can be used in two ways.
  - The adversary can initialize a protocol execution; sending the special message $M=\{U_{i_{1}},\ldots ,U_{i_{r}}\}\subseteq U$ to an unused instance $\Pi_{U}^{s}$ with U∈M initializes a protocol run among $U_{i_{1}},\ldots ,U_{i_{r}}$. After such a query, $\Pi_{U}^{s}$ sets ${\mathrm{pid}}_{U}^{s}:=\left\{U_{i_{1}},\ldots ,U_{i_{r}}\right\}$, ${\mathrm{used}}_{U}^{s}:=$ true, and processes the first step of the protocol.
  - The message *M* is sent to instance $\Pi_{U}^{s}$. The oracle returns the protocol message output by $\Pi_{U}^{s}$ after receiving *M*.
Reveal(U,s): returns the stored session key $sk_{U}^{s}$ if ${\mathrm{acc}}_{U}^{s}=$ true and a null value otherwise.
Corrupt(U): for a user U∈U this query returns *U*’s long-term secret key $ak_{U}$.

Notice that, unlike Reveal, Corrupt refers to a user instead of to an individual protocol instance.

We consider an adversary to be active if it has access to all of the above oracles. To capture a passive adversary, we can replace access to Send oracle with access to an Execute oracle, which returns a complete protocol transcript among the specified unused instances. An active adversary can simulate this Execute oracle using Send in the natural way.

For technical reasons, we introduce one more oracle, Test, and A must submit exactly one query of the form Test(U,s) with an instance $\Pi_{U}^{s}$ that has accepted a session key, i.e., with ${\mathrm{acc}}_{U}^{s}=$ true. In response to such a query, a bit b←{0,1} is sampled uniformly at random. For the case b=1, the established session key stored in ${\mathrm{sk}}_{U}^{s}$ is returned. For b=0 the output is a uniformly random element sampled from the space of session keys. The idea is that for a secure group key establishment protocol, no efficient adversary can distinguish between the cases b=0 and b=1. To turn this idea into a definition, we exclude trivial cases and focus on correct group key establishment protocols:

**Definition** **5** (Correctness)**.** *A group key establishment is said to be correct if on honest delivery of all messages and all users being honest, a single protocol execution among users $U_{0},\ldots ,U_{n-1}$ involves n instances $\Pi_{0}^{s_{0}},\ldots ,\Pi_{n-1}^{s_{n-1}}$ such that with overwhelming probability all of the following hold:*

- *all users accept, i.e., ${\mathrm{acc}}_{0}^{s_{0}}=\cdots ={\mathrm{acc}}_{n-1}^{s_{n-1}}=\mathrm{TRUE}$;*
- *all users obtain the same session identifier, i.e., ${\mathrm{sid}}_{0}^{s_{0}}=\cdots ={\mathrm{sid}}_{n-1}^{s_{n-1}}$;*
- *all users accept the same session key, i.e., ${\mathrm{sk}}_{0}^{s_{0}}=\cdots ={\mathrm{sk}}_{n-1}^{s_{n-1}}\neq \mathrm{NULL}$ associated with the same session identifier ${\mathrm{sid}}_{0}^{s_{0}}$;*
- *all communication partners are specified as desired communication partner, i.e., ${\mathrm{pid}}_{0}^{s_{0}}=\cdots ={\mathrm{pid}}_{n-1}^{s_{n-1}}=\left\{U_{0},\ldots ,U_{n-1}\right\}$.*

Correctness refers to a scenario where no attack takes place; to formulate security guarantees we need to specify the circumstances under which a correct guess for the random bit used by the Test oracle constitutes a possible attack. For this we use the following notions of partnering and freshness.

**Definition** **6** (Partnering)**.** *Two instances $\Pi_{U_{i}}^{s_{i}}$ and $\Pi_{U_{j}}^{s_{j}}$ are partnered if ${\mathrm{sid}}_{U_{i}}^{s_{i}}={\mathrm{sid}}_{U_{j}}^{s_{j}}$, ${\mathrm{pid}}_{U_{i}}^{s_{i}}={\mathrm{pid}}_{U_{j}}^{s_{j}}$, and ${\mathrm{acc}}_{U_{i}}^{s_{i}}={\mathrm{acc}}_{U_{j}}^{s_{j}}=$* true *.*

Based on this notion, we can determine what a fresh instance is, i.e., an instance where the adversary does not know the session key for trivial reasons.

The following formulation allows an adversary A to reveal all secret keys without violating freshness, provided A does not send any “relevant” messages afterwards. Therefore, security in the sense of Definition 8 below implies forward secrecy:

**Definition** **7** (Freshness)**.** *An instance $\Pi_{i}^{s_{i}}$ is called fresh if none of the following two conditions hold:*

- *For some $U_{j}\in {\mathrm{pid}}_{i}^{s_{i}}$ a $\mathrm{Corrupt}(U_{j})$ query was executed before a query of the form $\mathrm{Send}(U_{k},s_{k},*)$ has taken place where $U_{k}\in {\mathrm{pid}}_{i}^{s_{i}}$.*
- *A query $\mathrm{Reveal}(U_{j},s_{j})$ with $\Pi_{i}^{s_{i}}$ and $\Pi_{j}^{s_{j}}$ being partnered occurred.*

We write ${\mathrm{Succ}}_{A}^{\mathrm{ke}}$ for the event that A queries Test with a fresh instance according to Definition 7 and outputs a correct guess for the Test oracle’s bit *b*.

**Definition** **8** (Semantic security)**.** *A key establishment protocol is said to be semantically secure, if the advantage ${\mathrm{Adv}}_{A}^{\mathrm{ke}}=\left|2\cdot \mathrm{Pr}\left[{\mathrm{Succ}}_{A}^{\mathrm{ke}}\right]-1\right|$ is negligible for all probabilistic polynomial time algorithms A.*

To make explicit that adversaries are considered to be active, i.e., have access to the Send oracle, it is common to refer to a group key establishment protocol as authenticated. On the other hand, we speak of unauthenticated group key establishment if the adversaries are passive.

## 4. Proposed Construction

**Figure 3:**
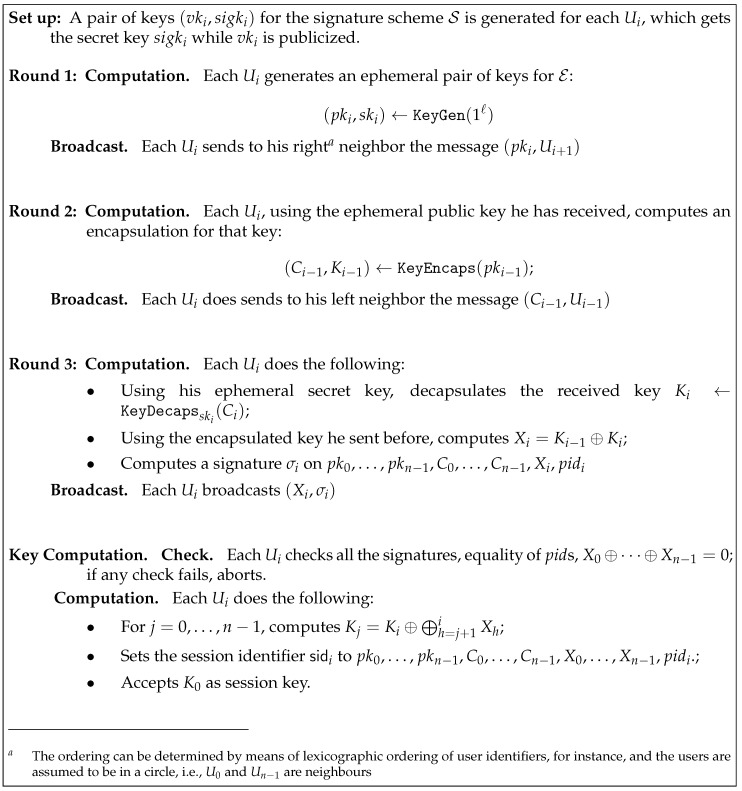
A compiler achieving authenticated group key establishment from a secure key encapsulation mechanism (KEM) and a secure signature scheme.

The proposed generic construction for designing an authenticated group key establishment for *n* users $U_{0},\ldots ,U_{n-1}$ is shown in Figure 3. It builds on an available key encapsulation mechanism and a signature scheme. The following theorem shows that the proposed construction offers a provable security guarantee, if the underlying primitives meet standard security requirements.

**Theorem** **1.** *Assuming S is an EUF-CMA secure signature scheme and E is an IND-CCA secure KEM, the protocol from Figure 3 is correct and semantically secure.*

**Proof.**  We can easily verify the protocol correctness: if all the participants follow the protocol description and there is no active adversarial interference, then all checks will succeed and every participant will set the same pid and sid. Moreover every participant will receive the correct ${\left\{X_{j}\right\}}_{j=0}^{n-1}$ and consequently they will be able to compute the same session key $K_{0}$.

To illustrate the security of our compiler, we use the “game hopping” technique, where we let the adversary A interact with a simulator B. We denote by $\mathrm{Adv}(A,G_{i})$ the advantage of the adversary in Game *i*. The security parameter is denoted by *ℓ*. Further, we denote by $q_{e}$ and $q_{s}$ the maximum number of queries made by the adversary to the Execute and Send oracles, respectively.

Game 0. This game is identical to the original attack game, with all the oracles being simulated as in the real protocol. Therefore,

$$\mathrm{Adv}\left(A,G_{0}\right)={\mathrm{Adv}}_{A}^{\mathrm{ke}}\left(\ell \right).$$

Game 1. Let Forge be the event that the adversary succeeds in forging an authenticated message $pk_{0},\ldots ,pk_{n-1},C_{0},\ldots ,C_{n-1},X_{i},pid_{i},\sigma_{i}$ of at least one party $U_{i}$ without having queried $\mathrm{Corrupt}(U_{i})$ and where all the values signed involved $pk_{0},\ldots ,pk_{n-1},C_{0},\ldots ,C_{n-1},X_{i},pid_{i},\sigma_{i}$ were not output by a same $U_{i}$’s instance. Any time this event occurs, we abort and mark this as success for the adversary.

An adversary A that can achieve Forge can be used to construct an adversary $A^{\prime }$ that forges a signature in the EUF-CMA game: the given public key is assigned randomly to $U_{i}$, one of the users; all other participants are initialized as the protocol indicates; afterwards all the queries in the security game are answered faithfully and when a signature by the chosen user is needed, the signing oracle of the EUF-CMA game is queried to produce it.

The probability of the adversary choosing $U_{i}$ when assigning the public key for the signature is at least 1/|U|, and with |U| being polynomial size, this is non-negligible:

$${\mathrm{Adv}}_{A^{\prime }}^{\mathrm{EUF}-\mathrm{CMA}}\geq \frac{1}{\left|U\right|}\cdot \mathrm{Pr}\left(\mathrm{Forge}\right),$$

which yields

$$|\mathrm{Adv}\left(A,G_{0}\right)-\mathrm{Adv}\left(A,G_{1}\right)|\leq {\mathrm{poly}}_{\mathrm{Forge}}\left(\ell \right)\cdot {\mathrm{Adv}}_{A^{\prime }}^{\mathrm{EUF}-\mathrm{CMA}},$$

for a polynomial bound ${\mathrm{poly}}_{\mathrm{Forge}}$ on |U|.

Game 2. This game is exactly as Game 1 except that a session *t* is chosen uniformly at random. If the Test query does not occur in the *t*-th session the game aborts, and we count it as win for the adversary. As the number of active protocol instances is polynomially bounded, we have

$$\mathrm{Adv}\left(A,G_{1}\right)\geq {\mathrm{poly}}_{\mathrm{Test}}\left(\ell \right)\cdot \mathrm{Adv}\left(A,G_{2}\right),$$

for a polynomial bound ${\mathrm{poly}}_{\mathrm{Test}}$ on the number of protocol sessions activated.

Game 3. This game is identical to the previous game, except that the simulation of the Send, Execute and Test oracles is modified as follows. The symmetric key $K_{0}$ output by $\mathrm{Encaps}(pk_{0})$ in the Test instance $\Pi_{0}^{t}$ is replaced with a random key $K^{*}$ chosen from ${\left\{0,1\right\}}^{\ell }$.

In order to bound the difference in the advantages between Games 2 and 3, we will build, from an adversary A ble to distinguish between both games, an adversary B attacking the key encapsulation mechanism E such that

$$|\mathrm{Adv}\left(A,G_{2}\right)-\mathrm{Adv}\left(A,G_{3}\right)|\leq 2\cdot {\mathrm{Adv}}_{B}^{\mathrm{IND}-\mathrm{CCA}}\left(\ell \right),$$

where ${\mathrm{Adv}}_{B}^{\mathrm{IND}-\mathrm{CCA}}\left(\ell \right)$ denotes the advantage of a probabilistic polynomial time adversary B attacking KEM. To establish this bound, we assume that B, which runs A as an auxiliary algorithm, can access a simulation of KEM. Further, B executes the key generation algorithm of S for each user $U_{i},$ thus obtaining a pair of keys $\left(vk_{i},sigk_{i}\right)$ for the signature scheme. Adversary B also executes the key generation algorithm of KEM for user $U_{0},$ and obtains the public key corresponding to users $U_{1},\ldots ,U_{n}$. Our adversary B obtains a challenge $\left(C^{*},K_{0},K_{1}\right)$ as described in Definition 1, and we have to describe how B answers to A’s queries:

- Whenever a query $\mathrm{Corrupt}(U_{i})$ is made by A, B generates the keys for the signature and returns $sigk_{i}$ as answer to A.
- To answer a Send query for Round 2 involving $U_{0}$, B uses the challenge encapsulation $C^{*}$. The rest of the answers are generated as in a real execution of the protocol.
- To answer an Execute query by A, our adversary B modifies the messages as described for the simulation of the Send oracle.
- A Reveal query by A is answered in a similar way as a Send or Execute query. Notice that a Reveal query cannot be made on *t* or any partnered instance. To answer any other Reveal query B uses the decapsulation oracle of its IND-CCA game.
- Finally, to answer a Test query, a bit $b^{\prime }$ is chosen by B when starting the simulation. B will return the key $K_{b^{\prime }}$ received from the KEM challenger to A.

At some point A will output a bit $b^{\prime \prime }$ as a guess for $b^{\prime }$ which will determine the output *b* of B for the KEM challenge. Specifically, B outputs b=0 if and only if $b^{\prime }=b^{\prime \prime }$. Taking into account that the view of A is identical to Game 2, if the answers of B’s simulation of Test are real keys and to Game 3 if the answers of B’s simulation of Test are random ones, we obtain that $|\mathrm{Adv}\left(A,G_{2}\right)-\mathrm{Adv}\left(A,G_{3}\right)|$ is bounded by $2\cdot {\mathrm{Adv}}_{B}^{IND-CCA}$.

To conclude the proof, we can see that the advantage of the adversary in Game 3 equals 0, as the session keys are chosen uniformly at random in ${\left\{0,1\right\}}^{\kappa_{\ell }}$. Collecting all the advantages, we can see that ${\mathrm{Adv}}_{A}^{\mathrm{ke}}$ is indeed negligible. □

### 4.1. Remark

We would like to point out that our scheme, as described above, meets precisely the security criteria described in Section 3. It would be possible to adjust our construction to capture additional security notions, for instance introducing contributory properties. In this case, only a minor modification becomes necessary. In fact, since all of $K_{0},\ldots ,K_{n-1}$ are available to each legitimate user, one could choose $K_{0}⊕\cdots ⊕K_{n-1}$ as session key, rather than just $K_{0}$. The proof would then be amended accordingly, as follows: in Game 3, after replacing $K_{0}$ with $K^{*}$, produce a pseudorandom session key as $K^{*}⊕\cdots ⊕K_{n-1}$. The pseudorandomness follows immediately from the fact that $K^{*}$ is chosen uniformly at random in ${\left\{0,1\right\}}^{\ell }$.

### 4.2. Instantiation

To instantiate the above AGKE construction, there are various natural options both for KEMs and signature schemes. Some possible candidates for quantum-safe KEMs include [14,15,16,17]. Moreover, examples for candidates of post-quantum signature schemes are offered by [9,18]. The particular choice of schemes can be made taking into account their efficiency or the assumption their security relies on. Different applications may have different preferences for prioritizing, e. g., public-key size over signature size.

## 5. Conclusions

The protocol compiler presented here offers a convenient approach to systematically design authenticated group-key establishment protocols in a post-quantum scenario. Keeping the number of signatures needed low (one per user), gives a protocol designer flexibility in the type of post-quantum signature signature scheme to be deployed. With three rounds, the round complexity that is achievable with currently available key encapsulation mechanisms appears quite attractive, too.
